# Editorial: Diabetic ketoacidosis in children and adolescents: From epidemiological data to clinical aspects

**DOI:** 10.3389/fped.2023.1164946

**Published:** 2023-03-09

**Authors:** Stefano Zucchini, Riccardo Bonfanti, Riccardo Schiaffini, Stefano Passanisi, Giuseppina Salzano, Fortunato Lombardo

**Affiliations:** ^1^ Pediatric Endocrine Unit, IRCCS Azienda Ospedaliero-Universitaria di Bologna, Bologna, Italy; ^2^ Pediatric Diabetes, San Raffaele Scientific Hospital and Vita Salute San Raffaele University, Milan, Italy; ^3^ Diabetes Unit, Bambino Gesú Childrens’ Hospital, Rome, Italy; ^4^ Department of Human Pathology in Adult and Developmental Age “Gaetano Barresi”, University of Messina, Messina, Italy

**Keywords:** diabetes, pediatric, glucose, insulin, incidence, diabetic ketoacidosis

**Editorial on the Research Topic** Diabetic ketoacidosis in children and adolescents: from epidemiological data to clinical aspects

Diabetic ketoacidosis (DKA) is a potentially life-threatening complication that usually occurs in at least one-third of newly diagnosed patients with type-1 diabetes (T1D) (1). Less frequently, it can occur in subjects with established diabetes because of insulin omission, interruption of insulin delivery in children on pump, or inadequate management of an infection (secondary DKA). The diagnosis of DKA is typically based on the triad of hyperglycemia, ketosis, and metabolic acidosis. Marked hyperglycemia and absence of ketosis characterize the hyperglycemic hyperosmolar state, which is rare in children with T1D and more typical of adults with T2D.

DKA occurs mainly because of the lack of insulin production as a consequence of β-cell destruction and a parallel increase of counterregulatory hormones induced by stress, such as catecholamines, glucagon, cortisol, and growth hormone. Dehydration is, in fact, inevitably associated with DKA and is caused by osmotic glycosuria with water and electrolyte loss. Most children presenting with DKA are in a volume-depleted state, resulting in acute tubular necrosis if it is a severe state; this may lead to acute kidney injury (AKI) (1). It is well known that the early detection of symptoms suggestive of T1D is pivotal for a prompt diagnosis and prevention, in order to avoid possible complications, prolonged hospital stays, excessive costs, and poor long-term metabolic control (2). DKA is associated with a wide range of complications, among which cerebral edema is the most feared, with a mortality rate that can go up to 24%, along with DKA-related cerebral injury. In addition to cerebral and renal derangements, other complications include severe electrolyte disorders, deep venous thrombosis, pulmonary embolism, rhabdomyolysis, and others.

DKA frequencies range from 15% to 70% in Europe and North America and DKA is typically more frequent in countries where T1D is less frequent (3). Young children are at an increased risk of presenting with DKA. DKA frequency at the onset of T1D has often been associated with the quality of the National Health Services offered. DKA awareness campaigns have proved effective in reducing the frequency of DKA at the clinical onset of type 1 diabetes in children and adolescents (4), but they require commitment and considerable economical effort on the part of healthcare providers and stakeholders.

Several countries have reported an increase in the frequency of DKA at the diagnosis of T1D in the last few decades, and recently, Birkebaek et al. (4) found a significant increase in the prevalence of DKA at the diagnosis of T1D in the years 2006–2019, with a marked additional increase observed during the COVID-19 pandemic in 2020 and 2021. It has been hypothesized that, above all, during the first wave of the pandemic, necessary initiatives such as social distancing and restricted non-essential services limited the access to healthcare services.

The present issue of Frontier in Pediatrics deals with important issues related to DKA in children with T1D, such as characteristics at diagnosis and possible complications.

The manuscript by Al-Abdulrazzaq et al. deals with the trends in the presentation of DKA in the years 2011–2017. Kuwait was among the countries with high diabetes incidence (40.9/100,000 children per year), and similarly, most countries of the world experienced a significant increase in newly diagnosed cases over the last 30 years. The study revealed that approximately one-third (35.9%) of children presented with DKA at diagnosis, and thankfully, only 9.1% were classified as severe. The good news is that compared with the year 1990, DKA incidence decreased significantly (it was 49%), although it was stable after the year 2000: the encouraging low number of cases was probably the consequence of both improvement in medical care in Kuwait and the fact that the increase in diabetes incidence in children, which almost doubled in the last 30 years, may have increased awareness on the alarming symptoms of the disease at onset. In contrast, the bad news is that the rate of severe DKA in younger children seems unchanged after the year 2000. However, the authors themselves report that some of the discrepancies found in DKA incidence are partly due to the establishment of the Childhood-Onset Diabetes electronic Registry in 2011 that may have impacted the results.

Interestingly, a study from a T1D low-incidence country (China) (Li GH et al.) showed that the DKA incidence rate was 52.7%. Compared with the non-DKA group, the DKA group showed a significantly lower age, a lower body mass index (BMI) z score, and higher antibody positive rates and HbA1c levels. The rate of incidence in patients younger than 5 years was as high as 56.7%. This was not explicitly reported in the study, but given the large case series (over 1,000 children younger than 5 years with DKA) and the rate of mortality of DKA (up to 20% in younger children), it was possible that quite a number of children were at risk for death and other serious complications associated with DKA. As expected from a country like China, T1D incidence was low (3.16/100,000/year), but similar to other Eastern countries, it showed a progressive increase. The authors underline the fact that the post-admission use of insulin pumps was lower (5.5%) in China than in European countries, the United States and Australia and attribute this problem to the poor availability of healthcare resources and patient apathy to treatment.

The case report by Frontino et al. well describes a rare case of mesenteric ischemia in a 13-year-old girl in the course of secondary DKA, caused by poor management of an insulin pump. The clinical presentation was serious, with hypovolemic shock, pH 6.9, hyperosmolar state, and AKI. Despite prompt resolution of the DKA, progressive abdominal pain persisted, and intestinal perforation was suspected: an intraoperative diagnosis of non-occlusive mesenteric ischemia was made. Vascular mechanisms associated with hypovolemia and shock seem to represent the main pathological mechanism involved in its genesis. This complication is thankfully rare in children, while it is more frequent in adults older than 50 years. Unfortunately, patients on pumps are more exposed to secondary DKA if they present psychological/psychiatric or social problems. The reported case, although examined at a high-level healthcare center, Italian Centre of Pediatric Diabetology, highlights the need for conducting an intensive surveillance on patients at risk, especially during adolescence. It is always necessary to educate patients and their families on a continuous basis in order to increase their levels of awareness on the issue of recognition of pump malfunctions and the subsequent appropriate treatment to be followed.

Finally, the study by Tinti et al. provided us an opportunity to describe the cases of three didactic patients and helped us detect and treat AKI occurring at T1D onset. In fact, despite severe acidosis and low bicarbonates, these patients unusually showed beta-hydroxy-butyrate (BOHB) levels below 3 mmol/L. The hypothesis was that acidosis probably had a composite origin from defective bicarbonate reabsorption in the kidneys and mild ketosis in the course of AKI. The authors underlined the usefulness of BOHB determination, which in this case, facilitated an understanding of the origin of acidosis, which could be attributed to tubular damage rather than to the classical DKA. On this subject, we suggest that all pediatric diabetologists, who are considered experts in diagnosing and treating AKI in the course of the initial dehydration that occurs and that is associated with a late diagnosis of T1D, to carefully read the recent systematic review on Pediatric Nephrology (5), which showed that one-fourth of DKA episodes were associated with a severe form of AKI requiring dialysis in 4% of patients. Both these cases and the previous one on mesenteric ischemia highlight the need to also consider the severe volume depletion that can be associated with DKA, so that a delicate balance could be achieved between addressing the risk of excessive fluid replacement, the known risk factor for cerebral injury, and the need to treat severe hypovolemia.

In conclusion, DKA remains a threatening condition for children with a late diagnosis of T1D or with an inappropriate management of insulin therapy all over the world. Despite the general improvement in the functioning of national health systems, the frequency with which DKA and its complications occur is unacceptably high. Poor tissue perfusion associated with DKA should be recognized and properly treated. On the one hand, all children should have the right to be treated in high-level pediatric centers that are equipped to treat DKA and AKI in accordance with the current guidelines. On the other hand, it is desirable that new strategies to prevent or at least delay the onset of T1D before the occurrence of DKA are devised at the earliest.
